# Indents

**Published:** 1910-02-15

**Authors:** 


### INDENTS.

**☞ Brief Articles of Technical or Scientific Value will receive notice and comment. Contributions invited.**

*A Dollar Earned versus A Dollar Saved.* A dollar saved is a dollar earned, with this difference, however: the saved dollar is a bird in the hand; you have it, and may set to work earning other dollars. The dollar earned is a bird in the bush. Although honestly earned, it is yours not till you get it. It may lead you a long chase; it may reach you clipped by a collector's fee. or tantalizingly flutter before you and finally land in the loss account. Nevertheless, inasmuch as earning is sowing, and saving harvesting, one must sow in order to reap. He who sows well has the best chance of harvesting abundantly. —Dr. Trueman in *Brief.*

*Medical Knowledge, its Acquisiton, and the M.D. Degree.* The fact that a dentist has not had the opportunity to attend a medical course and obtain an M.D. degree is no bar to his acquiring all the medical knowledge he finds necessary to fully compass his work in caring for the teeth and their surroundings. The possession of the M.D. degree is no help to a dentist who does not apply a knowledge of the collateral sciences in his daily work. Unless the M.D., D.D.S. keeps well abreast of the times, and well posted in the advances constantly being made in medical science, his medical diploma is a mere ornamental badge. The D.D.S. who is studious, who keeps well posted in all that pertains to his calling, has at command all that is required to do the utmost possible for his patient's good and his profession's advancement.—Dr. Trueman in *Brief.*

*Fees for service and not by Tradition.* During a conversation with a young dentist in my office regarding *professional business*, he remarked that—he tried to figure his fees by the hour for gold fillings but could not do it with the amalgam filling, as most of the patients who came to him were accustomed to paying only a dollar a piece for amalgam or cement fillings.

Now if there is any man in the profession who is *rendering professional services by exercising his best judgment in determining the most suitable remedy for restoring diseased or missing tissue to a state of health, comfort and usefulness*, who can give a logical reason why one remedy should be charged for by the hour and another by the piece, will he please make use of the pages of this journal and hasten to enlighten some of the benighted ones who are laboring under the impression that it is for the rendering of our *judgment*, *skill* and *services* that we should receive our fees and not for the sale of so much gold, amalgam, or cement, to be delivered at the discretion of the patient and at whatever price he may see fit to offer?—F. C. Brush, in *Digest*.

*Herpes and Canker Sores.* Dr. Gilmer referred to my belief that the so-called canker sores are manifestations of simple herpes in the mouth. I base that view on the ground that these lesions are not at all in their manifestations like simple infections. If any of you have had them, you know they are excessively painful and develop suddenly. These lesions have the acute pain and sudden onset which belongs to cold sores on the lips. The lesions occur when one is out of sorts, with indigestion, or with malaise of any kind. They may come from local irritation, as fever blisters come from local irritation. But their intense painfulness and their sudden development point to their being a manifestation similar to herpes about the lips. I believe that fever sores and canker sores of the mouth are due to a neuritis in the peripheral terminals of the affected area, which may be set up by toxemia, other sys temic disturbances or by local irritation, such as riding in a cold wind, or the irritation from dental instruments.—Dr. Pusey in *Review*.

*Risky Investments; a Caution.* A recent letter repeats that often-heard error: "A man with only a few thousands must make more than 6 per cent., for what would 6 per cent. on so small a sum amount to? A man with $50,000 or $100,000 can afford to take a low rate of interest, because it will amount to anough to be worth while, and will make him comfortable." I wish I could strike this error such a blow that it would never be heard of again. The man with only a few thousands saved must continue to work; but in the meantime he should guard with special jealousy the safety of that which he has already saved. What is the use of saving if one is going to be reckless with it and lose it? A man is two kinds of a fool if he expects to stop work, put on airs and live on his investments after he has saved only a few thousands. He is foolish to think that he can do so, and he is foolish to put his precious savings into such necessarily unsound investments as those are which promise returns equal to a moderate rate on a larger amount of safe investments.—Editorial, "Business Talk to Doctors," *The Medical World*, April, 1909.

*Where They Come From.* "Where do you get your patients?" is frequently asked. From every source; surely, this is a charity of charities. They come from the Orphan Asylums, Protestant, Catholic and Jewish. The various Hospitals, The Home of the Friendless, The Children's Aid Society, The Society for the Prevention of Cruelty to Children, The Industrial School, The Door of Hope, the Health Association, the Health Bureau, The Tuberculosis Hospital, The Day Camp; every Public and Parochial School in the city. From ministers, Priests, Physicians, Teachers, Dentists and Laymen. An effort is made to investigate every case, many of the children do not need investigating; their poorly nourished bodies speak for themselves.

> "That all softening, overpowering knell
> The tocsin of the soul—the dinner bell."

has been conspicuous by its absence. They come to the Clinic and look up to you with big trustful eyes and are grateful and appreciative. Surely, these are God's Poor!

*Anaesthetic Death Rate in England and Wales.* Recently compiled statistics, brought out in the discussion of a bill now before the British Parliament, depriving qualified dentists of the right to administer anaesthetics for tooth extraction and like operations, shows an alarmingly high death rate from anaesthetics. The figures given are for all anaesthetics, including nitrous oxid.

| | |
|---|---|
| 1903 | 146 deaths |
| 1904 | 156 " |
| 1905 | 155 " |
| 1906 | 183 " |
| 1907 | 186 " |

Of these 826 deaths during five years, thirteen only are credited to nitrous oxid. Quite a large number of these fatal cases have occurred in the dentist's chair, the anaesthes tic having been administered by a medical practitioner, a- the right of a dentist not medically qualified to administer an anaesthetic is questioned. The present bill seems to prohibit a dentist not medically qualified from administer-ing any anaesthetic, local or general, for any purpose what-ever. The figures for 1908 are not yet available. This high death rate is largely due to the medical anaesthetist not appreciating the fact known to all dentists, that more care is needed when a patient is seated in a chair than when lying on a bed or operating table. Another factor is the more general use of chloroform.—*Dental Record* (London) June, 1909, page 367.

*Is it Worth While?* On the first presentation of the little patient, they are relieved of their immediate sufferings, and if needed, the teeth are given a prophylactic treatment. The child is asked if they have a tooth brush and powder; if they have not, they are presented with both and instucted in their use and the teeth are expected to be clean on their next visit; if not, they are warned that they will be sent home. If the child has dirty face and hands, they are told the Dentist cannot work for them until they are clean. They are introduced to a cake of soap and a towel in an adjoining room. These children soon learn, and present themselves with improved conditions. The lessons are carried home and must have an influence on the family life. Quite a number of the applicants are the children of our foreign population. We are helping to make them better citizens, better men and women; the care of the teeth is a step toward the care of the body in general, and with it increased confidence and self-respect.

In our city, recently, as the result of a family brawl, six children were thrown on the world and came under the care of the Children's Aid Society. The eldest, a girl of 14 years, had been the little Mother of the family. "Why are the children so dirty?" she was asked. "Why, they ain't dirty; they had their faces and hands washed every week," was the reply.

What do you think would have been the effect had this little girl come under the influence of our Dispensary and been taught its lesson of cleanliness?—*Dispensary Record.*

*The Need of Free Dental Services.* In a series of articles by Robert Grant, that appeared in Scribner's Magazine, a number of years ago; he describes a certain stage of poverty as follows: "You know the kind of family I mean; the one to whom the Dentists bill is an unexpected calamity."

Yes, we know them; nice respectable people with numerous children; the heads of which are denying themselves the little comforts of life to educate their offspring. They have a stated income and have made provisions for clothes schooling, the baker, butcher and the coal man; life insurance, entertainment, even the chance of sickness—but they have left the Dentist out of their calculation and *his* bill *is* an unexpected calamity! To pay it, they have to pinch somewhere; perhaps the wife goes without a new dress or a much needed hat; does it cheerfully and no one is the wiser. God bless those mothers of ours! Perhaps YOU had one who pinched and saved that her children might have an education. Talk about MARTYRS! And some people say that because such mothers—yea fathers—didn't have time or were too proud to go to church and parade their poverty—they did not go to heaven! You and I know better—most people do now-a-days.

What are we doing for the children of the worthy poor who are sadly in need of dental services? It requires no argument that a family of six, with a total income of $10 per week and paying $3.50 house rent, cannot afford the luxury of a Dentist. To the children of the worthy poor, brought into the world without their consent; poorly nourished, without sufficient clothing to cover them decently; with a widowed mother or a consumptive father as a bread winner; it is a duty and a privilege to help. We may not give a tenth of our income—but have we a right to give—NOTHING!—*Dispensary Record.*

*History of Dental Dispensary.* The first Free Dental Dispensary in the United States, for that matter in the world as far as we know, was established by the Rochester Dental Society, some twenty-five years ago, in the Rochester City Hospital. This, after a trial of two years was abandoned and it was not till a period of twenty years had passed that the matter was again taken up by the society. In view of the former failure, the older members were unwilling to again embark on a similar undertaking.

Some of the younger members, in view of the alarming need of dental services among our worthy poor, persisted in the feasibility of the idea and a committee was appointed; after numerous reports of nothing accomplished, it was announced that the late Capt. Lomb would donate a sum of money sufficient to equip a Free Dental Dispensary.

It was decided to establish the charity at the Rochester Public Health Association, 32 South Washington Street. The Dispensary was opened to the public on Washington's Birthday, Feb. 22nd, 1905, and for the first year, twenty-four members of the society alternated in attendance. Capt. Lomb then offered to pay for the services of a Dentist who would be in attendance from 2 to 5 P. M. each week day. This arrangement continued to the day of his death; in fact, the family paid for this until the beginning of the present year. It became necessary to call on the general public for support and it was with a feeling of their inefficiency that the Board of Directors took up this work. It was determined to send out a personal letter to the charitably inclined and in it give a statement of our work and the needs of the coming year.

As a result of this appeal, the sum of $1,387.50 was secured. This amount was sufficient to carry on the work but not enough to increase its scope as we had hoped.—*Rochester Dental Dispensary Record.*

*The Dentist and Charity.* The Dentist, as an individual, is doing all he can—he gives generously of his time and talents as far as a busy office practice will permit, but he has not the time or the opportunity to dispense charity outside of his office. The physician is in a different class and there are several good reasons for this: The Dentist has long office hours and is expected to keep them; his labors are continuous and exhausting if he is to make more than a mere living. On the other hand, the physician has short office hours—if he is not in his office outside of these hours, it is rather to his credit, as he is supposed to be busy. He makes four calls or forty and has some portion of the day for charity and prepares himself for the FIFTIETH, for a patient who is willing and can afford to pay for *experience.*

The Dentist cannot do much charity work in an hour or two; nor is he adding to his skill or fame, in performing operations embracing no greater field than amalgam and cement fillings, cleaning teeth or extracting.

In the case of a number of Dentists, one following another, in attendance for the day only, it is impossible to avoid a certain amount of duplication of work among those patients whose work cannot be completed in one sitting. This is not for the best interest of the Dispensary or the patient; some such condition is created as might be with a medical case of, say, scarlet fever, where the first medical attendant prescribed, and on the following days there would be a new medical adviser with an honest difference of opinion of the treatment of the case, and consequent confusion and probable death of the patient, who might have done well under the treatment of any one of the gentlemen.

To avoid this confusion and absence of corelation in the services rendered, we have found it better to have one practitioner, or at most two, in attendance who would give this work their undivided attention and receive compensation for his services.—*Dispensary Record.*

*Free School Dispensaries Abroad.* Germany has led the world in this work and established a large number, some say a dozen are in operation, another authority says there are over fifty cities so equipped. One of the latest is the City of Berlin; the Berlin authorities had the matter under consideration for some time, and other institutions will shortly follow. The treatment of the very poor will be gratis; parents whose means will permit will be asked to pay twelve cents annually. The most elaborate of these institutions is situated in the City of Strasburg, where they have erected a $60,000 building for the Dental Dispensary. This action of the German authorities was taken after the alarming fact was ascertained that over 80 per cent. of the teeth of her school children were defective and the indirect cause of many ills. In the third annual report of the City of Strasburg, of 2,269 children examined between the ages of three and four only 362 had good teeth—less than 16 per cent. Of 2,103 between the ages of six and eight, only 160 or seven and one-half per cent. and this third report shows a remarkable improvement in general health of the public school children; less headache, earache and stomach troubles. What must have been the condition there prior to the compulsory education in the care of the teeth!

The German people are among the first to recognize the fact that the proper care of the teeth has a great influence on the general health. More and more, it has become a demonstrated fact, that no person's health can be better than his teeth. They realized that the health of their children were at stake and again, perhaps, the possibility of an army of soldiers without teeth! What use then of expensive army maneuvers and equipment; these must have been their reasons for making it compulsory to have the teeth of school children examined twice yearly and any who could not pay to be treated at public expense.

Even Russia, poor tax-burdened Russia; a country we consider as unprogressive, has many Free Dental Dispensaries for its poor school children. St. Petersburg with a population approximating that of Philadelphia, has *Nine* such Free Dental Dispensaries. Thus we see that even the heavily taxed countries of Europe have made such matters a Municipal charge, which is included in the general taxes. We of this country, unless we have made such things a special study, can have no conception of the burdensome taxes paid by these countries; people taxed to the utmost, and yet they think it necessary to add to their burdens. Free Dental Dispensaries for their poor school children! Surely there must be a reason!

